# Multi-Step Relaxation Characterization and Viscoelastic Modeling to Predict the Long-Term Behavior of Bitumen-Free Road Pavements Based on Polymeric Resin and Thixotropic Filler

**DOI:** 10.3390/ma17143511

**Published:** 2024-07-15

**Authors:** Carina Emminger, Umut D. Cakmak, Zoltan Major

**Affiliations:** Institute of Polymer Product Engineering, Johannes Kepler University Linz, Altenbergerstraße 69, 4040 Linz, Austria; umut.cakmak@jku.at (U.D.C.); zoltan.major@jku.at (Z.M.)

**Keywords:** bitumen-free asphalt pavement, linear elastic modeling, viscoelastic modeling, relaxation characterization, polymeric resin pavement, mineral filler, long-term asphalt behavior

## Abstract

Asphalt pavements are fundamental to modern transportation infrastructure, requiring elasticity, firmness, and longevity. However, traditional asphalt, based on bitumen, faces several limitations. To improve pavement performance, polymer resins are being used to substitute bitumen and improve requirements. Therefore, a deep understanding of the material behavior is required. This study presents the analysis of the relaxation behavior of a poly(methyl methacrylate)-based pavement and the influence of mineral fillers. An approach using a linear elastic–viscoelastic material model was selected based on evidence and validated across the linear and nonlinear deformation range. The results reveal no influence of the mineral fillers on the relaxation behavior. The presented modification of the linear elastic and viscoelastic modeling reveals accurate results to predict long-term pavement performance. This approach offers a practical method for forecasting asphalt behavior. Further research is needed to incorporate deformation behavior into the model.

## 1. Introduction

Asphalt pavements are critical components of modern transportation infrastructure, providing the necessary surface for vehicles to travel safely and efficiently. Traditionally, asphalt mixtures have relied on bitumen as a binder due to its adhesive and waterproofing properties [1]. However, bitumen-based asphalt presents several challenges, including limited processability at ambient temperatures [2,3] and significant environmental concerns due to its high global warming potential (GWP) [4]. These limitations have driven the search for alternative materials that can enhance the performance and sustainability of asphalt pavements.

In recent years, polymer resins have emerged as a promising substitute for bitumen in road pavements [1]. Polymeric materials such as styrene–butadiene–styrene (SBS) and ethylene–vinyl acetate (EVA) have proven to improve the durability and resistance of pavements while reducing their GWP [2,5,6,7,8]. Resin-based pavements offer a versatile foundation for customizing properties to meet specific needs. For example, their static friction can be adjusted to improve slip resistance in industrial areas. By incorporating reinforcing agents and other additives, the pavement’s elastic and thermoelastic properties can be tailored to ensure it fulfills the required performance standards for various applications [9]. In addition, the incorporation of thixotropic fillers has been shown to optimize the rheological properties of pavements, addressing issues such as segregation and flow during processing [10,11].

Despite these advancements, a comprehensive understanding of the viscoelastic behavior of polymer-based pavements is essential for predicting their long-term performance [12,13,14]. Moreover, the influence of processing and filler additives and how these can be used to modify pavements are of interest too (cf. [9,15,16]). The behavior of asphalt under various loads can be categorized into three distinct ranges: linear viscoelastic, nonlinear viscoelastic, and destructive [17]. Characterizing the viscoelastic properties, particularly the relaxation behavior, is crucial for developing reliable material models that can forecast the performance and longevity of asphalt pavements under real-world conditions. The standardized viscoelastic characterization is performed with dynamic experimental tests, which are specified by guidelines such as the ASHTO or ASTM specifications [18]. All define the dynamic mechanical complex modulus tests at specified temperatures and frequencies. A master curve can be created from these results, and material parameters can be derived from it for finite element (FE) calculations [19,20]. Bitumen-based and bitumen-free pavements exhibit viscoelastic behavior [15,21,22,23]. Viscoelastic modeling plays a pivotal role in pavement condition assessment, enabling the prediction of material responses to dynamic loads and environmental variations [24,25]. Bai et al. [26] used a viscoelastic model to predict the stress–strain response of asphalt pavements under nonuniform-distributed tire-pavement contact pressure. Asim and Khan [27] used uniaxial tensile stress-relaxation tests to model the viscoelastic behavior of asphalt concrete, whereas Ban et al. [28] conducted creep tests to obtain viscoelastic material properties of pavements. However, all material models were generated in the linear viscoelastic range at small deformations [19,21,24,25,27,28,29]. If higher deformations were considered, the models were extended to viscoplastic [30] or nonlinear viscoelastic behavior [24], which requires further experimental investigations. In this study, a linear elastic–viscoelastic approach for bitumen-free pavements is presented, generated from three-step relaxation tests, within and outside the linear elastic range.

**Figure 1:**
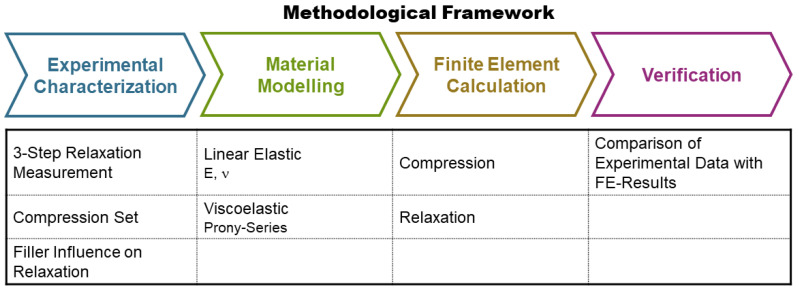
Methodological framework.

The objective of this study was to gain insights into the relaxation behavior of bitumen-free asphalt pavements and, specifically, the influence of three mineral fillers (i.e., basalt sand (BS), silica sand (SS), and silica dust (SD)). The methodological framework is presented in Figure 1. An experimental three-step compression relaxation measurement was performed (Experimental Characterization). From these results, the influences of the three mineral fillers on the relaxation behavior, as well as on the compression set, were investigated. Moreover, the experimental data were used to determine parameters for a material model, which can be used in numerical analysis (Material Modeling).

Regarding the material model, a linear elastic–viscoelastic approach was used. The linear elastic model was chosen for its simplicity, requiring only two parameters. The viscoelastic behavior was modeled with a Prony series with five parameters, according to the experimental relaxation measurement. The parameters for modeling the long-term behavior were generated and validated across the linear and nonlinear deformation range.

## 2. Materials and Methods

### 2.1. Materials

Within this study, seven different material formulations were selected to investigate the influence of three different mineral fillers on the relaxation behavior of bitumen-free asphalt pavements and determine parameters of a Prony series to model the linear viscoelasticity based on five elements of the generalized Maxwell model. A poly(methyl methacrylate) (PMMA)-based resin from Silikal GmbH (Mainhausen, DEU) was used and filled with a thixotropy agent (hydrophilic fumed silica with a specific surface area of 200 m^2^/g), a binder, a catalysator, and a color pigment to improve processability. The three mineral fillers were silica dust (SD), silica sand (SS), and basalt sand (BS). The difference between SD and SS lies in the particle size. Previous works [15,31] reveal an increase in compressive strength with a higher amount of SD, due to the smaller particle size. The total amount of mineral fillers was 80 wt% but with systematically varying composition for each formulation. The different formulations are presented in Table 1, and the bulk density [32,33,34] of each filler and processing agent is shown in Table 2. F2, F6, and F7 refer to dust-dominant formulations, whereas the remaining are named sand-dominant formulations.

The viscoelastic behavior of these materials and the optimum amount of filler were characterized in a previous study [15]. The materials were provided by RoadPlast Mohr GmbH (Vorarlberg, AUT), and the specimens were cast cylinders with ∅15 mm × 15 mm.

### 2.2. Methods

#### 2.2.1. Experimental Relaxation Test

**Figure 2:**
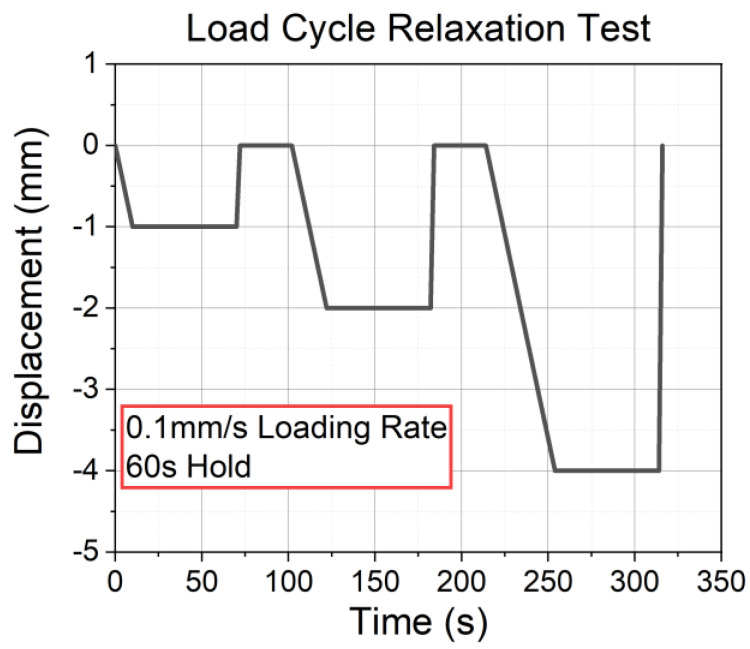
The procedure of the three-step relaxation test.

For the characterization of the relaxation behavior, three-step relaxation tests were performed. The measurement procedure is shown in Figure 2. The specimens were loaded while controlling displacement with 0.1 mm/s under compression at three different states: −1 mm, −2 mm, and −4 mm. Each position was held for 60 s, and after the relaxation time, the specimen was fully unloaded (back to 0 mm) within 30 s. The experiment was performed with a servo-hydraulic test system (MTS 852, MTS System Corporation, Eden Prairie, MN, USA) under isothermal conditions at 20 °C. The force was recorded with a 10 kN load cell (661.19F-02 Force Transducer-10kN, MTS System Corporation, Eden Prairie, MN, USA). The specimens had a cylindrical shape (∅15 mm × 15 mm). From the experiment, the relaxation behavior at different compression states, the compression set (CS), and the relaxation slope (k) of each material formulation were measured and analyzed.

The CS [%] was calculated according to Equation (1), where h_0_ is the original specimen height, h_i_ is the specimen height after testing, and h_n_ is the spacer thickness during the measurement. CS was calculated after the final compression step of 4 mm. So, $h_{n}=h_{0}-4$.

(1) $$CS=\frac{h_{0}-h_{i}}{h_{0}-h_{n}}\times 100$$

The relaxation slope k [s^−1^] was calculated according to Equation (2), where F_Comp_ is the load; t_Comp_ is the time after the compression at each compression level (−1 mm, −2 mm, and −4 mm); and F(t_i_) is the load after t_i_, which was set to 2 s. For better comparison, k was normalized to F_Comp_. The exact values are given in Section 3.

(2) $$k=\frac{1}{F_{Comp}}\times \frac{\Delta F}{\Delta t}=\frac{1}{F_{Comp}}\times \frac{F\left(t_{i}\right)-F_{Comp}}{t_{i}-t_{Comp}}$$

#### 2.2.2. Numerical Implementation

##### Material Modeling for Prony Series Parameters

To predict the relaxation behavior of each material formulation, Prony parameters were determined using MCalibration (MCalibration 7.2.6, Ansys, Canonsburg, PA, USA). Therefore, the *Abaqus Linear Elastic–Viscoelastic* material model was chosen, due to the simplicity of the linear elastic model, requiring only two parameters. The linear elastic model is used to simulate the compressive deformation, whereby it is considered that the materials do not reveal ideal linear elastic material behavior. The focus of this research is on the determination of the relaxation behavior of the materials, which is modeled with the generalized Maxwell viscoelastic (phenomenological) model. A Prony series with five parameters (Maxwell elements) was chosen for the modeling of the viscoelastic behavior.

**Figure 3:**
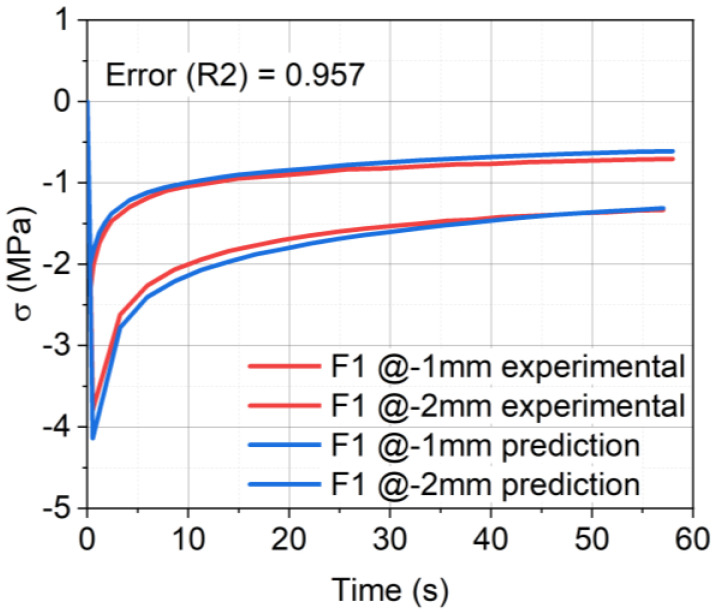
Linear elastic and Prony series fit of the experimental relaxation data at −1 mm and −2 mm compression, with error (R2), the coefficient of determination, and metric for understanding the proportion of variance fitted by the model.

The parameters were generated by fitting the experimental data from the relaxation tests of −1 mm and −2 mm compression, as depicted in Figure 3. The figure shows the experimental data (red curves) and the modeled data (blue curves). The error (R2, coefficient of determination) was calculated to analyze how well the experimental results were reproduced by the model. The Poisson’s ratio (ν) was set to 0.3 in all cases. According to the work of Aurangzeb et al. [35], ν was used in the range from 0.25 to 0.35 for asphalt pavements. Gonzalez et al. [36] also used 0.3 as the value for ν. Additionally, 0.3 was chosen because it aligns with commonly accepted values found in related studies, ensuring consistency and comparability of results. It is important to note that the value of ν set to 0.3 is only valid at 20 °C. It has to be adjusted accordingly due to the high-temperature dependence of ν [37,38]. The determined parameters for the *Abaqus Linear Elastic–Viscoelastic* model of the material formulation F1 are given in Table 3. The values for F2–F7 are given in Table A1, Table A2, Table A3, Table A4, Table A5 and Table A6 in Appendix A.

According to the results presented in Section 3, the material formulation is no longer linear at a deformation of −4 mm. This is due to the higher deformation compared to −1 mm or −2 mm. In contrast, the material was pre-stressed and compacted by the first two measurement cycles, leading to a higher compressive set. For the specified material models, these two reasons lead to large deviations in the calculation of F_Comp_. To apply the linear elastic–viscoelastic model to the results of the −4 mm compression tests, Young’s modulus has to be adjusted by a factor γ, as shown in Equation (3). The predicted moduli of each formulation, the adjusted moduli, and the adjusting factor γ are shown in Table 4. γ was retrieved by dividing the E_Adjusted_ with E, and prior E_Adjusted_ was fitted linear with the experimental data from −4 mm compression.

(3) $$E_{Adjusted}=\gamma \times E$$

###### Virtual Setup

The finite element simulation was performed using Abaqus 2020 (Abaqus CAE, Dassault Systems, FRA). A *solid* cylinder with dimensions of ∅15 mm × 15 mm was created and meshed using an 8-node linear brick (C3D8) with a mesh size of 0.6 mm. The clamp was modeled as *discrete rigid* with a diameter of ∅20 mm, using a 4-node 3D bilinear rigid quadrilateral (R3D4) mesh with a size of 2.0 mm. The material properties of the specimen were modeled as *Elastic* and *Viscoelastic*, with parameters as described previously. Prior to the performed simulations, a mesh-sensitivity analysis was performed to optimize the mesh size regarding the calculation time of the simulation.

To model the experimental setup, the cylinder was positioned between two clamps: one at the bottom, constrained with the boundary conditions encastre, and another at the top to apply the compressive deformations of −1 mm, −2 mm, and −4 mm. The interaction between the clamps and the cylinder was modeled as *surface-to-surface* contact, with the clamp as the *master surface* and the specimen as the *slave surface*. A tangential behavior with an estimated coefficient of friction of 0.3 was specified as the *contact interaction property*.

The simulation was executed in two sequential steps: first, compression was applied using a *static*, *general* procedure, and subsequently, relaxation was simulated for a duration of 60 s using *visco* analysis.

###### Numerical Verification

**Figure 4:**
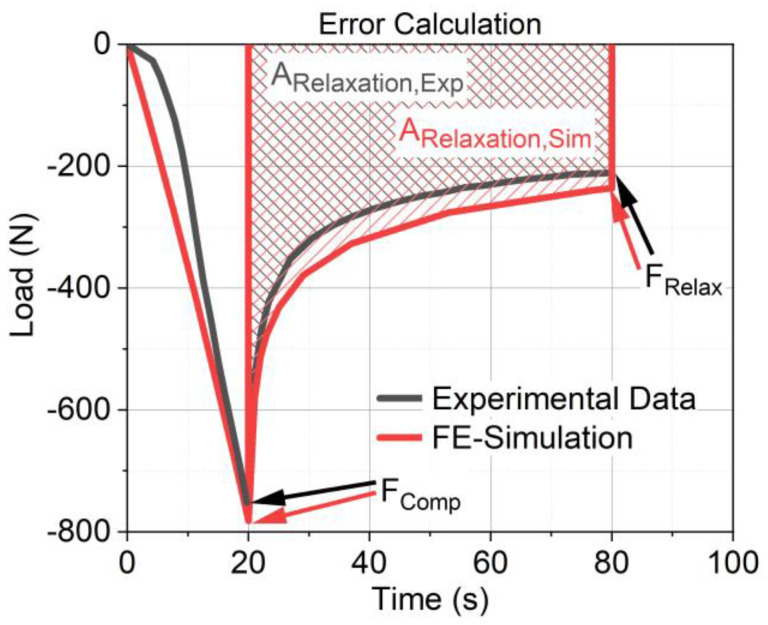
Illustration of the error calculation of the whole relaxation time.

To validate the results of the FE simulation, three values of the experiment and the simulation were compared. The errors are shown in Figure 4. First, the load at the end of the compression (F_Comp_) was determined. The calculation of F_Comp_ in the numerical analysis should be as accurate as possible because it is the beginning of the relaxation calculation. High deviations at this stage cause follow-up errors in the prediction of relaxation. In the second step, the load at the end of the relaxation (F_Relax_), and in the third, the total relaxation (A_Relaxation_) were determined, where the relaxation data were integrated over the entire relaxation time, and the deviation in area between the experimental and simulation data was determined. Since one of the objectives was to predict the relaxation behavior, a low deviation from the FE-calculated F_Relax_ from the experimentally measured F_Relax_ was targeted. Moreover, not only was the value at the end of the numerical analysis of interest but also the modeling of the whole relaxation process. Therefore, the A_Relaxation_ of the experiment was compared with the A_Relaxation_ of the FE calculation. Accurate modeling allows the relaxation to be calculated at any time within 60 s.

The relative error (ER) of all three parameters was calculated according to Equation (4), where x_SIM_ is a variable for F_Comp,SIM_, F_Relax,SIM_, or A_Relaxation,SIM_ from the simulation results, and x_EXP_ is a variable for F_Comp,EXP_, F_Relax,EXP_, or A_Relaxation,EXP_ from the experimental data.

(4) $$ER\;\left[\%\right]=\frac{x_{SIM}-x_{EXP}}{x_{EXP}}\times 100$$

## 3. Results

### 3.1. Results of the Experimental Relaxation Measurements

**Figure 5:**
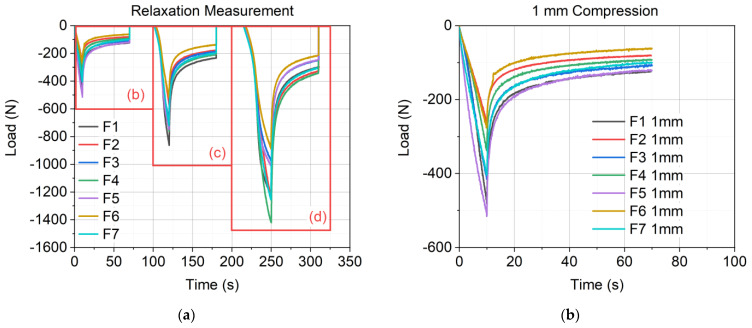

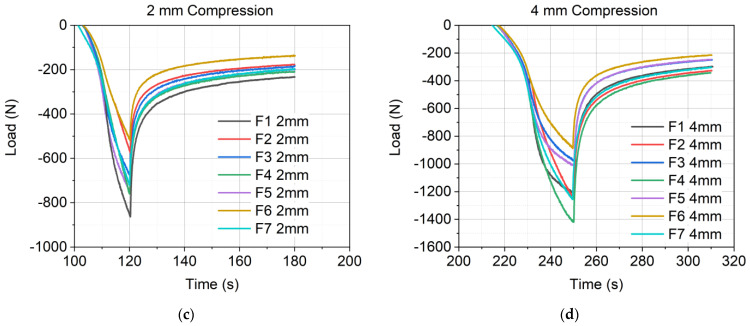
Results of the experimental relaxation measurement: (**a**) relaxation data of the whole measurement cycle; (**b**) relaxation data of all formulations at the first compression step at −1 mm; (**c**) relaxation data of all formulations at the second compression step at −2 mm; (**d**) relaxation data of all formulations at the third compression step at −4 mm.

The experimental results of the three-step relaxation measurements are shown in Figure 5. Figure 5a reveals the material response of all seven material formulations at each compression step, and Figure 5b,c display the relaxation behavior of all formulations at one compression step from −1 mm to −4 mm.

**Figure 6:**
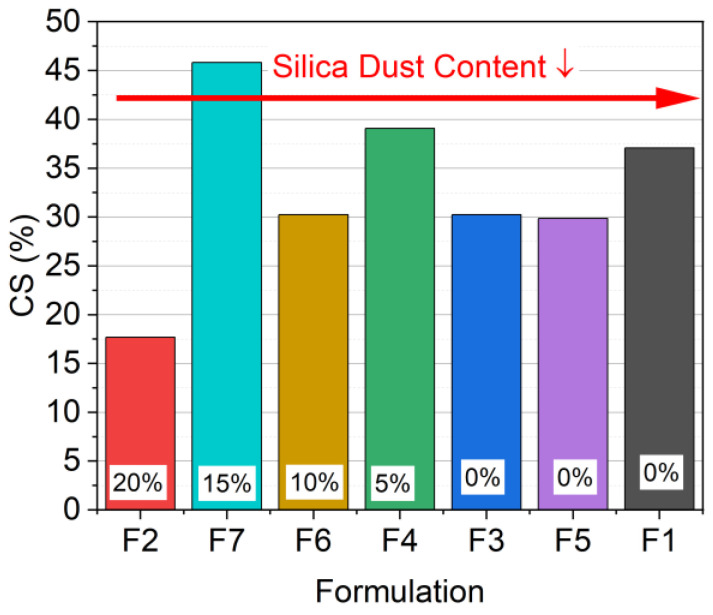
Compression set (CS) of all material formulations after 73.5% compression.

Figure 6 shows the results of the compression set (CS) in order of a decreasing amount of SD and an increasing amount of sand, as well as a decreasing amount of SS.

**Figure 7:**
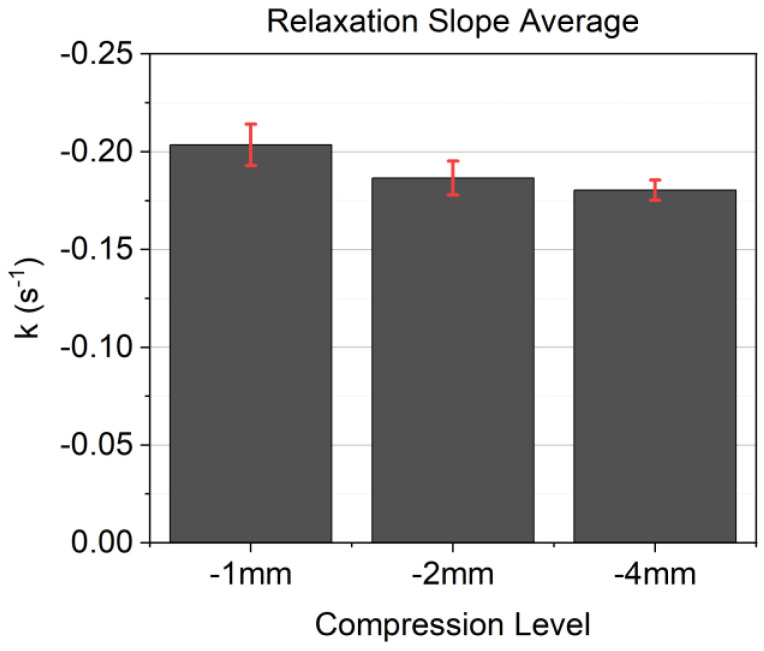
Average of the relaxation slope k of all three compression levels.

Figure 7 shows the averages of the relaxation slope k at all compression levels, including the ±standard deviation. Additionally, Table 5 lists the exact values of the results, the averages, and the standard deviation.

### 3.2. Results of the Numerical Analyses

**Figure 8:**
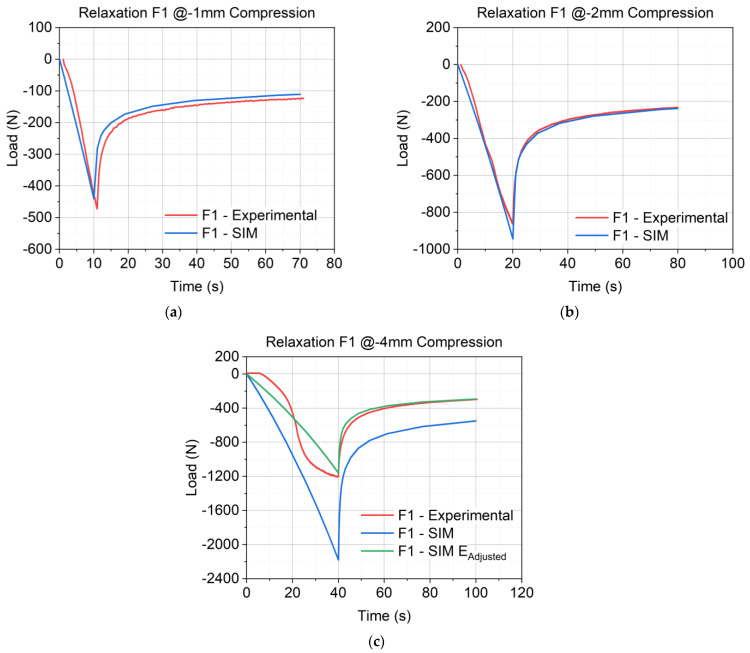
Results of the FE simulation of material formulation F1: (**a**) results at −1 mm compression; (**b**) results at −2 mm compression; (**c**) results at −4 mm compression; the blue curve shows the results with the same E as for −1 mm and −2 mm, and the green curve presents the results with the adjusted modulus.

**Figure A1:**
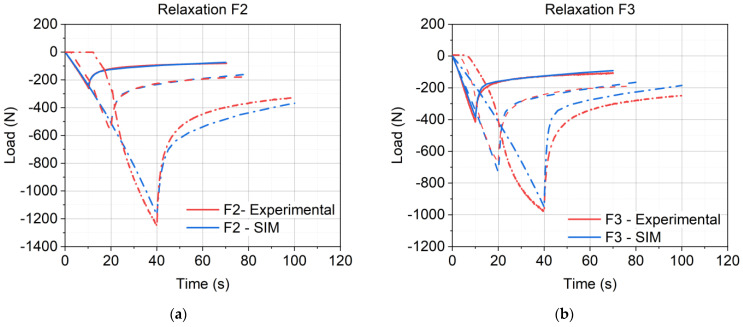

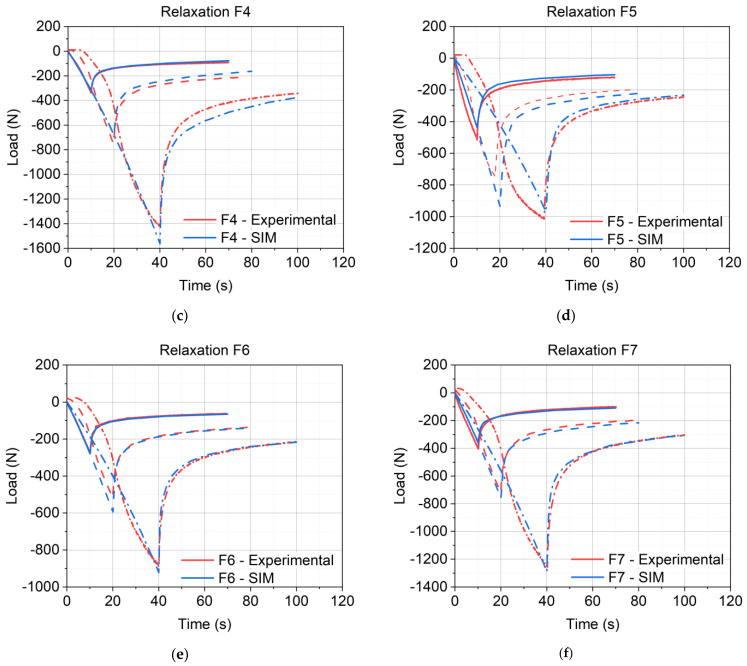
Results of the FE calculations for all three load cases. For the −4 mm compression, the adjusted modulus was used: (**a**) F2, (**b**) F3, (**c**) F4, (**d**) F5, (**e**) F6, and (**f**) F7. The solid lines represent the −1 mm, the dashed line the −2 mm, and the dash-dotted line the −4 mm compression.

Figure 8a–c illustrate the results of the finite element (FE) simulation at each compression state for material formulation F1. The results for F2–F7 are presented in Appendix A in Figure A1a–f. Figure 8c presents the results of the simulations with the same modulus used in Figure 8a,b and the adjusted modulus. Further, it highlights the necessity to adjust the moduli to generate good predictions. As shown in Table 4, the E_Adjusted_ of F1 is only 53% of the original modulus E.

### 3.3. Results of the Verification

**Figure 9:**
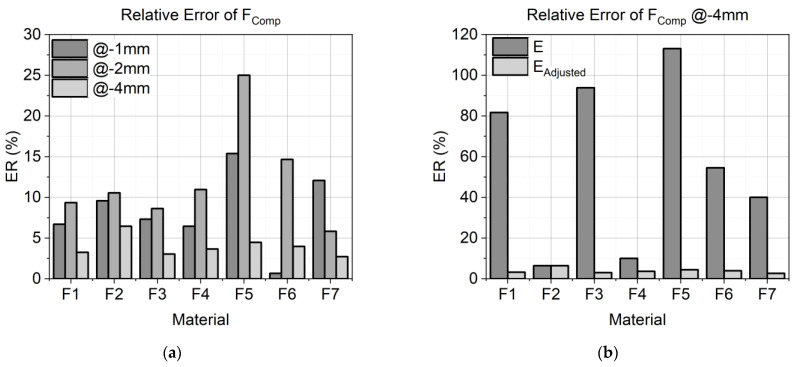
Error calculation (ER) of the experimentally determined load at the end of the compression and the FE calculated one for each material formulation: (**a**) error of F_Comp_ at all compression states of all material formulations with E_Adjusted_ at −4 mm; (**b**) error of F_Comp_ at −4 mm in comparison of the modulus E, used for −1 mm and −2 mm, and E_Adjusted_.

The calculated relative errors of the compression load (ER of F_Comp_) between the experimental and the FE-calculated load of all material formulations are presented in Figure 9a. For each material formulation, all ER values for each compression state (−1 mm, −2 mm, and −4 mm) are presented in graduated shades of gray. The errors presented at the −4 mm compression state were calculated with the results of the adjusted modulus (E_Adjusted_). Figure 9b illustrates the high deviation of F_Comp_ of all materials at −4 mm between modulus E values, fitted for −1 mm and −2 mm compression and E_Adjusted_. Due to the better results with E_Adjusted_ at −4 mm compression, in what follows, the values only show the results obtained with E_Adjusted_ and no longer refer to the results achieved with E.

**Figure 10:**
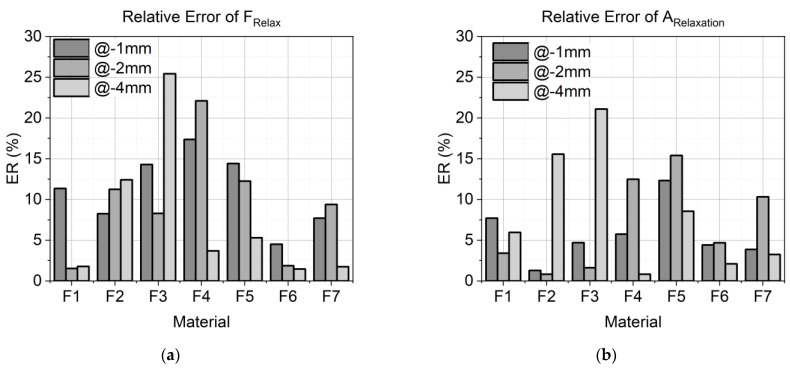
(**a**) Error calculation of the experimentally determined load at the end of the relaxation and the FE-calculated one for each material formulation at each compression state; (**b**) error calculation of the experimentally determined relaxation area A_Relaxation,Exp_ and the FE-calculated one A_Relaxation,Sim_ for each material formulation at each compression state.

Figure 10a shows the calculated relative error (ER) of the relaxation load F_Relax_ between the experimental load and the FE-calculated load of all materials for the compression at −1 mm, −2 mm, and −4 mm in shades of gray. Furthermore, the relative error of the areas (ER of A_Relaxation_) of the total relaxation time for all seven material formulations is presented in Figure 10b for all three compression states.

## 4. Discussion

The results of the three-step relaxation measurement, shown in Figure 5a–d, reveal a similar relaxation behavior for all seven material formulations at each compression level (−1 mm, −2 mm, and −4 mm). This correlation is also shown in the mean values of the relaxation slope k in Figure 7, which leads to the expected conclusion that regarding the relaxation behavior of the investigated materials, their polymeric matrix is the origin for viscoelasticity, and the mineral fillers have no influence on the relaxation behavior. Furthermore, k exhibited a decrease with an increase in compression from −1 mm to −2 mm but stayed constant for −2 mm and −4 mm. The work of Liu et al. [39] shows that relaxation decreases after passing the yield point in glassy polymers in tension and compression. This states a decrease in k for the higher compression at −2 mm and −4 mm. However, the relaxation behavior of the materials was similar (see Figure 5), and the compression behavior showed a more nonlinear behavior with an increased compression state. As shown in Figure 5b, all material formulations show a linear behavior, whereas in Figure 5c, F5, F6, and F7 already show a nonlinear behavior. Proceeding to the higher compression level, as shown in Figure 5d, only F2 and F4 remain linear, whereas all other formulations show pronounced nonlinearity. The increase in nonlinearity with an increase in compression was already studied in [15], which revealed that the material formulations with a higher amount of SD (also referred to as dust-dominant formulation) show a stiffer behavior compared to sand-dominant formulations. Dust-dominant formulations reveal a more linear compression behavior too, whereas sand-dominant materials show a broader load-carrying plateau.

The examined compression set (CS), shown in Figure 6, points to the influence of mineral fillers. A significant difference in the CS was observed for all material formulations. The results indicated that formulations with greater variation in the filler particle size had greater variation in the CS. F7 and F4 were the only formulations that contained both SD and SS and, therefore, had the highest variation in particle size and the highest CS. This finding led to the conclusion that the more particles of the same size, the more constant the value of the CS. This was observed in F3 (20 wt% SD: 60 wt% BS), F5 (10 wt% SS: 70 wt% BS), and F6 (10 wt% SD: 70 wt% BS). The results also showed that a higher number of small particles led to a lower CS than larger particles (F2 (20 wt% SD: 60 wt% BS) vs. F1 (80 wt% BS)).

The fitted material models reveal good simulation results. Figure 8a–c show the experimental data in comparison to the results of the simulation for F1. For −1 mm (Figure 8a) and −2 mm (Figure 8b), the compression behavior as well as the relaxation behavior were reproduced with high accuracy. This is also shown in the low values of relative error of F_Comp_ in Figure 9a (6.7% at −1 mm and 9.3% at −2 mm), the relative error of F_Relax_ in Figure 10a (11.4% at −1 mm and 1.6% at −2 mm), and the error of A_Relaxation_ in Figure 10b (7.7% at −1 mm and 3.4% at −2 mm). For higher compression states, the compression behavior was found to be far too stiff, as shown in Figure 8c. Hence, the implementation of γ to adjust modulus E was necessary to predict good results. With the adjusted modulus E_Adjusted_, the prediction of F_Comp_ is possible with good quality, as shown in Figure 9b. Only F2 and F4 would have predicted good results without E_Adjusted_, due to their highly linear behavior. This is also demonstrated by the high values of γ (F2 1.00 and F4 0.88), whereas high nonlinear formulations require low γ values like F1 and F5 (0.53 and 0.45). Comparing the error of F_Comp_ in Figure 9a, it can be inferred that all material formulations revealed low deviations between the experimental and the FE-calculated load values, except for F5, which showed high deviations for the results of −1 mm and −2 mm (15.4% and 25%). As F5 has a lower content of SD, a significant nonlinearity is observed at −2 mm (see Figure 5c), and the fitted modulus is an average of both compression states and, therefore, shows higher deviations. As mentioned above, F6 and F7 behaved nonlinearly at −2 mm too, which exhibited a higher deviation from −1 mm (0.7%) to −2 mm (14.7%) for F6. Interestingly, F7 showed a higher deviation for −1 mm (12.1%) compared to −2 mm (5.8%). The modeling fitted Young’s modulus for both load cases in one step, and in this case, it fitted the second load case better than the first.

To conclude on the relaxation behavior, an exact calculation of F_Comp_ is required, because it is the beginning of the relaxation calculation and can cause follow-up errors. Figure 10a illustrates the error of F_Relax_ for all material formulations, while Figure 10b depicts the error of A_Relaxation_. Interestingly, no direct correlation was observed between the error of A_Relaxation_ and the error of F_Relax_. Using the material parameters, the relaxation load F_Relax_ F_Relax_ could be predicted with a deviation of 15% from the experimental results for most materials and load cases. However, formulations F3 and F4 exhibited higher deviations. F3 had an error of 26% at −4 mm compression. The relaxation behavior was also modeled using experimental data from −1 mm and −2 mm compressions. Due to reduced relaxation at −4 mm, indicated by a decrease in the relaxation time constant with increased compression, the model requires further refinement to yield accurate predictions. F4 showed an error of 17% for −1 mm and 22% for −2 mm compressions. For −4 mm compression, the error was below 5%. According to the decreased relaxation time constant at higher compression levels, the calibrated model predicted too low relaxation for small deformations.

The deviation in A_Relaxation_ was below 5% for more than half of the materials and load cases, with only 5 out of 21 cases revealing deviations higher than 10%. Notably, F2 exhibited a 16% deviation at −4 mm, and F3 showed a 22% deviation at −4 mm. These higher deviations are due to the variation in relaxation at higher compression levels, as previously discussed. F4, with a 13% deviation at −2 mm, reflected higher deviations in F_Relax_, while F5, with deviations of 13% at −1 mm and 15% at −2 mm, indicated follow-up errors from the high deviations of F_Comp_ at −1 mm and −2 mm compressions. However, F_Relax_ results for these formulations still showed a deviation below 15%.

## 5. Conclusions

To conclude, the results of the experimental investigations revealed that mineral fillers have no influence on the relaxation behavior of the different material formulations but influence the stiffness of the compression behavior. They also affected the compression set (CS) according to particle sizes and their distribution; with higher content of smaller particles (SD), a lower CS was observed and vice versa. Further, in formulations with more different particles, the CS revealed higher values than for formulations with only one or two types of particles.

The investigated linear elastic–viscoelastic approach and the used material models reveal good results with respect to F_Comp_, F_Relax_, and A_Relaxation_, which enables a prediction of the relaxation behavior and the long-term behavior of the different material formulations. The results highlight the applicability and the limitations of the linear elastic model. For low-compression deformations, the linear elastic–viscoelastic approach fits the compression behavior as well as the relaxation behavior. For higher deformations, the linear elastic model reveals stiff results, but the limitations can be extended with the adjusted modulus and the required adjusting factor γ. This enables an exact prediction of the compression force for higher nonlinear deformations. However, in the current study, the compression behavior was not exactly reproduced with the adjusted version.

These results highlight an understanding of the composition and influence of mineral fillers in bitumen-free asphalt pavements. The presented modeling approach enables an uncomplicated parameter fitting, which can be used for numerical analysis to predict the composition of material formulations for desired mechanical properties in the application. However, compressive deformation is only accurately modeled in the linear elastic range. Further investigations are required for a detailed simulation of compression in the nonlinear range. Moreover, the material model can be extended by damage hypotheses, but these require additional experimental studies.

## Figures and Tables

**Table 1:** Material formulations with the amount of fillers.

Material Formulation	Silica Dust (SD) in wt%	Silica Sand (SS) in wt%	Basalt Sand (BS) in wt%	Ratio Dust–Sand
F1	0	0	80	0:80
F2	20	0	60	20:60
F3	0	20	60	0:80
F4	5	5	70	5:75
F5	0	10	70	0:80
F6	10	0	70	10:70
F7	15	15	50	15:65

**Table 2:** Fillers and processing agents and their bulk density.

Filler/Processing Agent	Bulk Density [g/cm^3^]
Silica dust	2.65
Silica Sand	2.65
Basalt sand	2.71
Thixotropy agent	2.2
Pigment	4.6
Catalysator	0.62
Binder	0.98

**Table 3:** Prony parameters of F1 from the relaxation measurement using the FE software Abaqus 2020.

Parameters	g_i_	k_i_	τ_i,t_	E	ν
Units	-	-	s	MPa	-
1	0.36044	0.3504	0.50	33.8	0.3
2	0.15096	0.1910	3.23		
3	0.10181	0.0952	8.62		
4	0.05211	0.0524	22.06		
5	0.11754	0.1163	46.30		

**Table 4:** Young’s moduli (E) and adjusted Young’s moduli (E_Adjusted_) of all material formulations.

Material Formulation	E [MPa]	E_Adjusted_ [MPa]	Γ [-]
F1	33.80	18.00	0.53
F2	18.17	18.17	1.00
F3	29.50	14.75	0.50
F4	24.24	21.22	0.88
F5	33.46	15.00	0.45
F6	21.26	14.30	0.67
F7	25.44	20.00	0.79

**Table 5:** Calculated relaxation slope k at all compression levels.

Material Formulation	k @−1 mm [s^−1^]	k @−2 mm [s^−1^]	k @−4 mm [s^−1^]
F1	−0.2045	−0.1995	−0.1734
F2	−0.1864	−0.1747	−0.1727
F3	−0.2043	−0.1925	−0.1816
F4	−0.2017	−0.1888	−0.1830
F5	−0.2174	−0.1887	−0.1855
F6	−0.2144	−0.1857	−0.1846
F7	−0.1956	−0.1765	−0.1817
Average	−0.2035	−0.1866	−0.1804
Standard deviation	±0.0106	±0.0087	±0.0052

## Data Availability

The original contributions presented in the study are included in the article, further inquiries can be directed to the corresponding author.

## Appendix A

In the following tables (Table A1, Table A2, Table A3, Table A4, Table A5 and Table A6), the parameters generated with MCalibration for the material formulations F2 to F7 are presented. The used model was the *Abaqus Linear Elastic–Viscoelastic*.

**Table A1:** Prony parameters of F2 from the relaxation measurement for the FE software Abaqus.

Parameters	g_i_	k_i_	τ_i,t_	E	ν
Units	-	-	s	MPa	-
1	0.3496	0.3498	1.79	18.17	0.3
2	0.0336	0.0762	4.95		
3	0.0313	0.0374	5.91		
4	0.1966	0.1797	49.69		
5	0.2019	0.1612	51.69		

**Table A2:** Prony parameters of F3 from the relaxation measurement for the FE software Abaqus.

Parameters	g_i_	k_i_	τ_i,t_	E	ν
Units	-	-	s	MPa	-
1	0.0242	0.8845	0.40	29.50	0.3
2	0.0025	0.0032	3.92		
3	0.0181	0.0014	28.55		
4	0.7255	0.0263	61.78		
5	0.2297	0.0550	118.15		

**Table A3:** Prony parameters of F4 from the relaxation measurement for the FE software Abaqus.

Parameters	g_i_	k_i_	τ_i,t_	E	ν
Units	-	-	s	MPa	-
1	0.2580	0.3458	0.70	24.24	0.3
2	0.2387	0.2394	3.26		
3	0.0683	0.0921	25.62		
4	0.1015	0.0840	36.68		
5	0.1558	0.1232	56.35		

**Table A4:** Prony parameters of F5 from the relaxation measurement for the FE software Abaqus.

Parameters	g_i_	k_i_	τ_i,t_	E	ν
Units	-	-	s	MPa	-
1	0.3441	0.3441	1.51	33.46	0.3
2	0.2069	0.2069	2.83		
3	0.1125	0.1125	11.94		
4	0.0583	0.0583	52.44		
5	0.1008	0.1008	60.17		

**Table A5:** Prony parameters of F6 from the relaxation measurement for the FE software Abaqus.

Parameters	g_i_	k_i_	τ_i,t_	E	ν
Units	-	-	s	MPa	-
1	0.2956	0.3558	0.3	21.26	0.3
2	0.2379	0.2364	2.8		
3	0.1619	0.1543	16.7		
4	0.0737	0.0117	48		
5	0.0235	0.0730	56		

**Table A6:** Prony parameters of F7 from the relaxation measurement for the FE software Abaqus.

Parameters	g_i_	k_i_	τ_i,t_	E	ν
Units	-	-	s	MPa	-
1	0.0065	0.7679	0.30	25.44	0.3
2	0.0024	0.0742	2.81		
3	0.0023	0.0854	15.46		
4	0.5398	0.0001	63.72		
5	0	0.0002	93.33		

In Figure A1a–f, the results of the FE calculations are presented for all three load cases and compared to the experimental data. The presented results for −4 mm compression were calculated with the adjusted modulus. The red curves present the experimental values, and the blue curves show the calculated data. The solid lines show the compression at −1 mm, the dashed line shows the compression at −2 mm, and the dash-dotted line shows the compression at −4 mm.
